# Supplementary figures and images for: Integrated Proteotranscriptomics Reveals Differences in Molecular Immunity between Min and Large White Pig Breeds
Source: Biology (Basel). 2022 Nov 25;11(12):1708. doi: 10.3390/biology11121708 (PMC9775064; doi:10.3390/biology11121708)

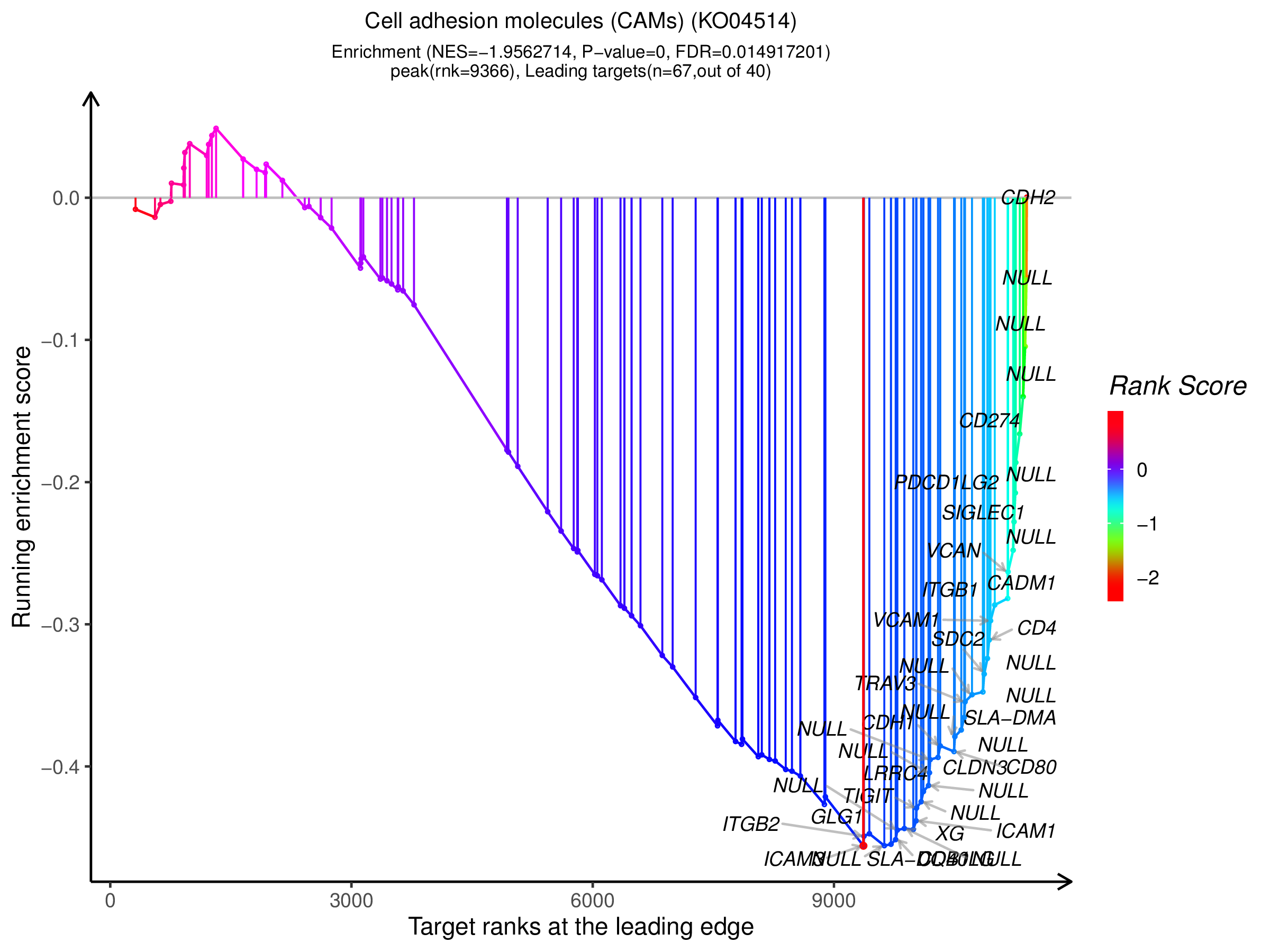

Supplement: Supplementary file 1 [file biology-11-01708-s001.zip › Supplementary Figure S1.png]

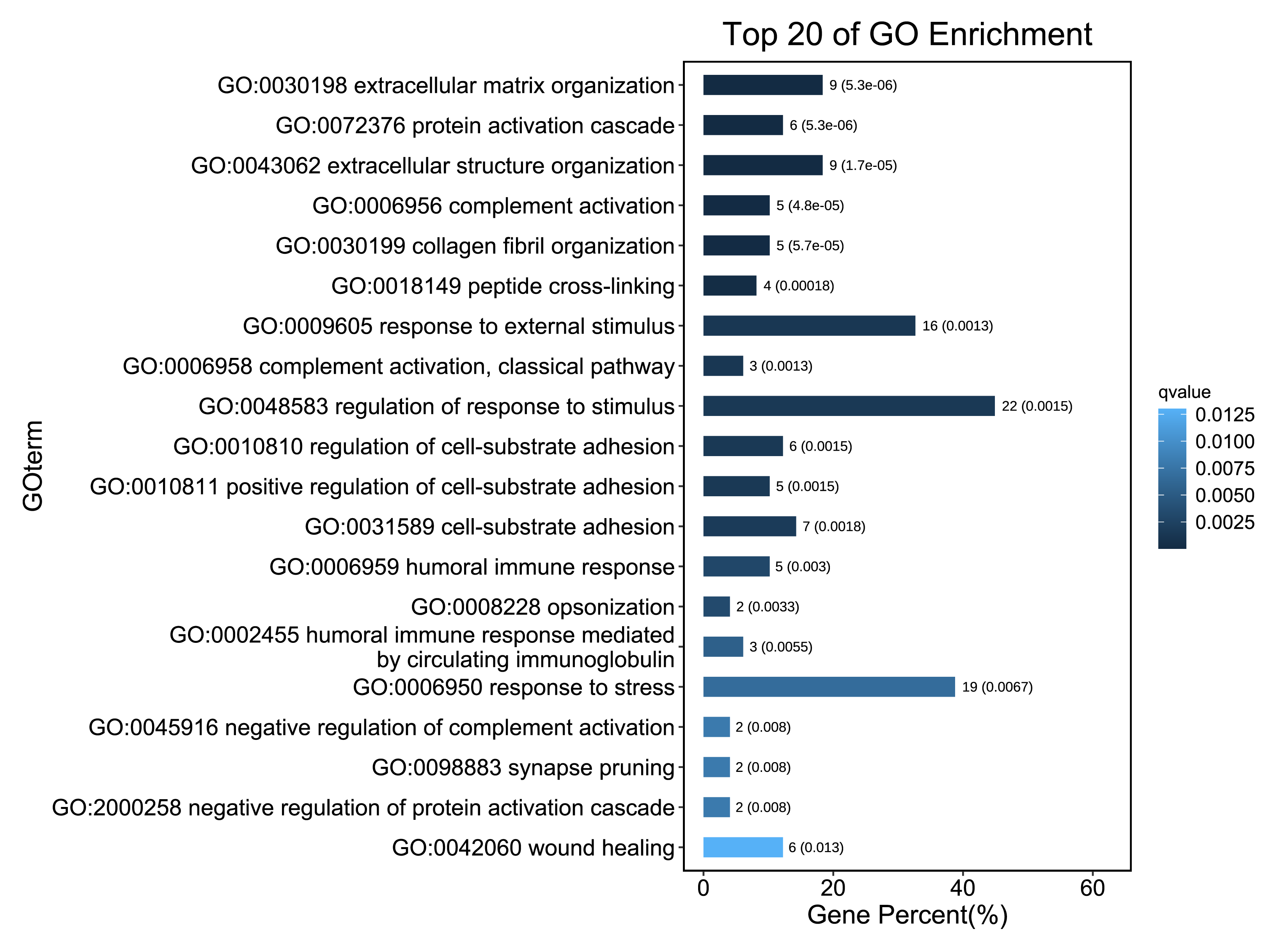

Supplement: Supplementary file 1 [file biology-11-01708-s001.zip › Supplementary Figure S2.png]

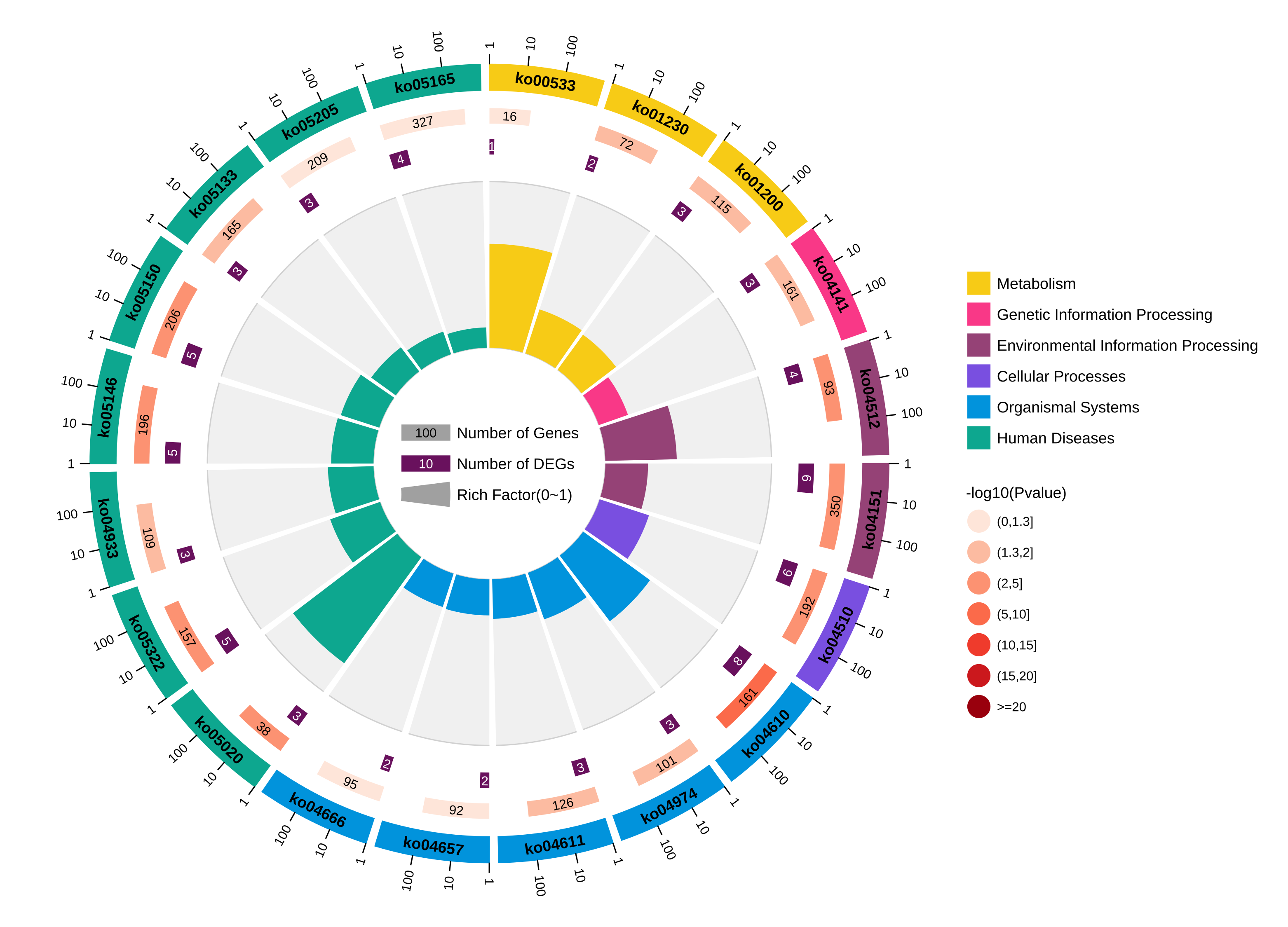

Supplement: Supplementary file 1 [file biology-11-01708-s001.zip › Supplementary Figure S3.png]

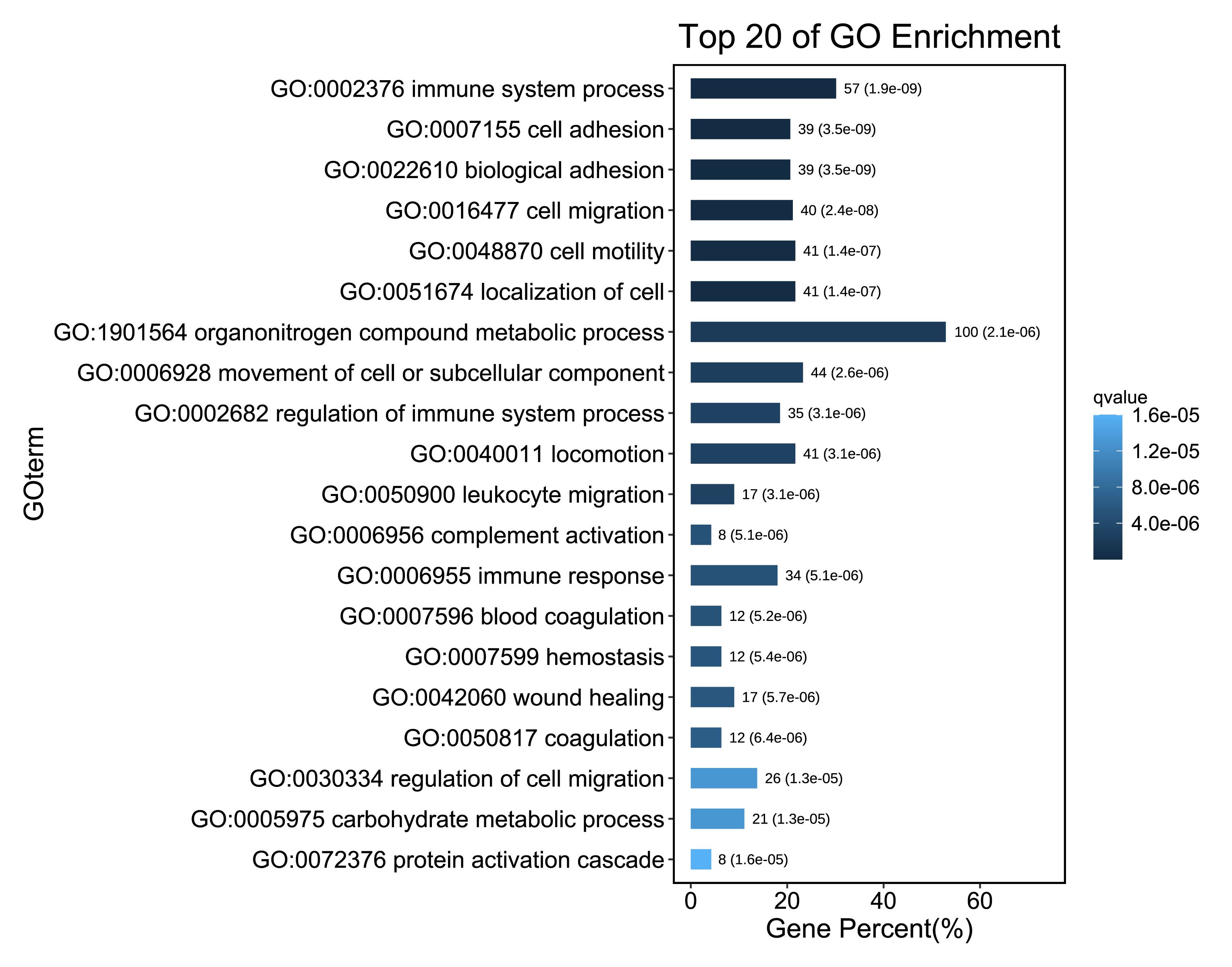

Supplement: Supplementary file 1 [file biology-11-01708-s001.zip › Supplementary Figure S4.png]

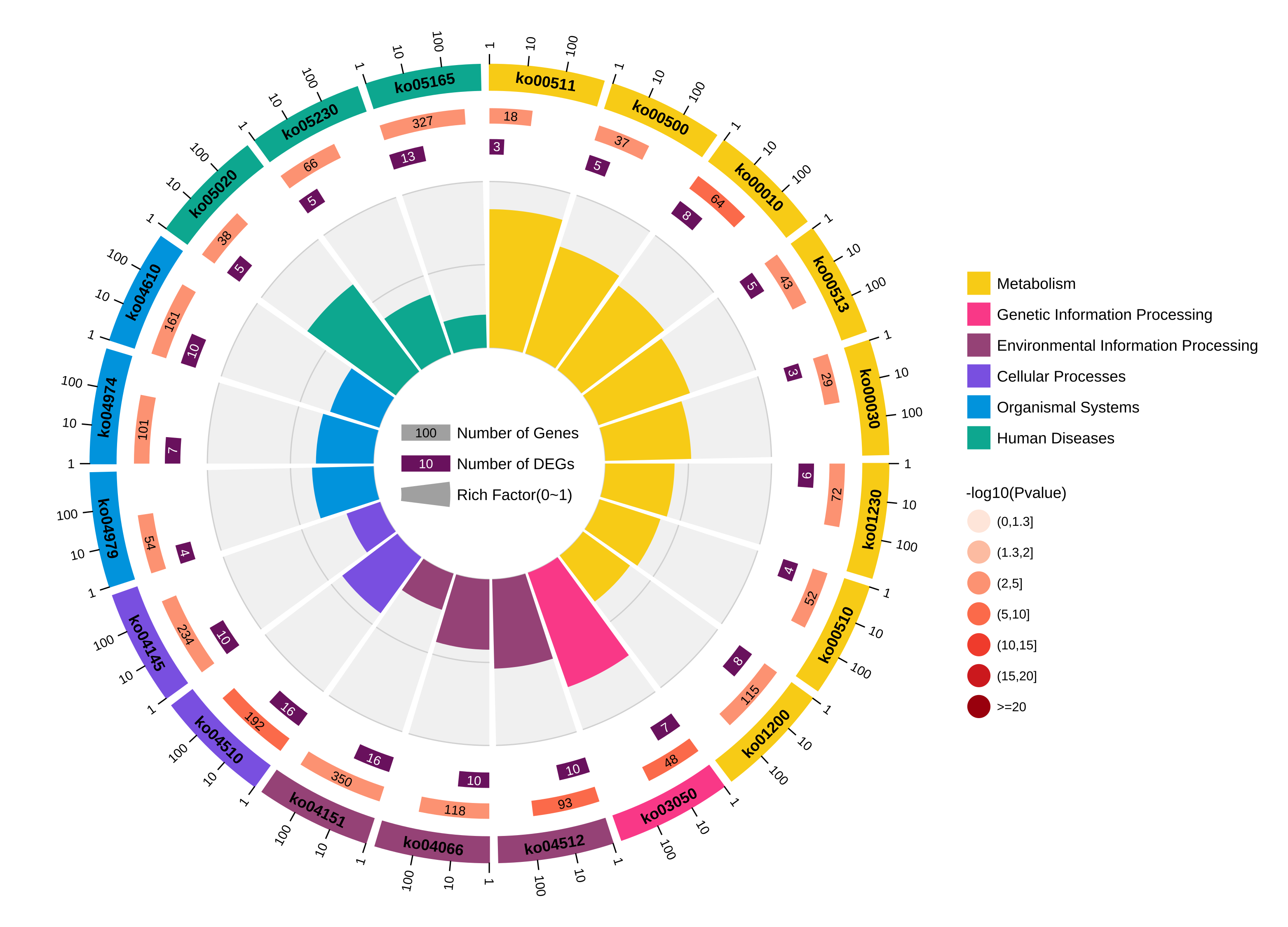

Supplement: Supplementary file 1 [file biology-11-01708-s001.zip › Supplementary Figure S5.png]

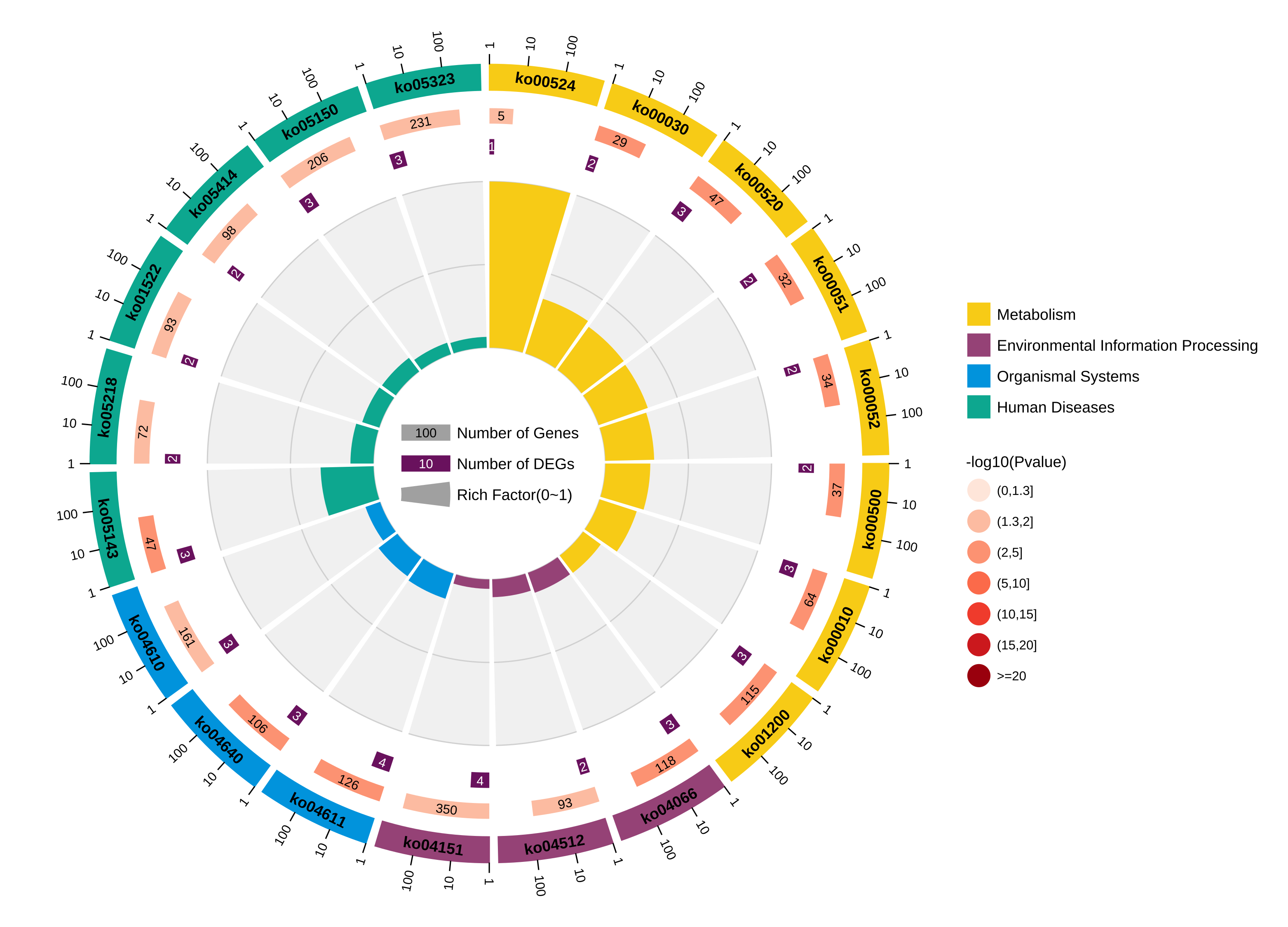

Supplement: Supplementary file 1 [file biology-11-01708-s001.zip › Supplementary Figure S6.png]

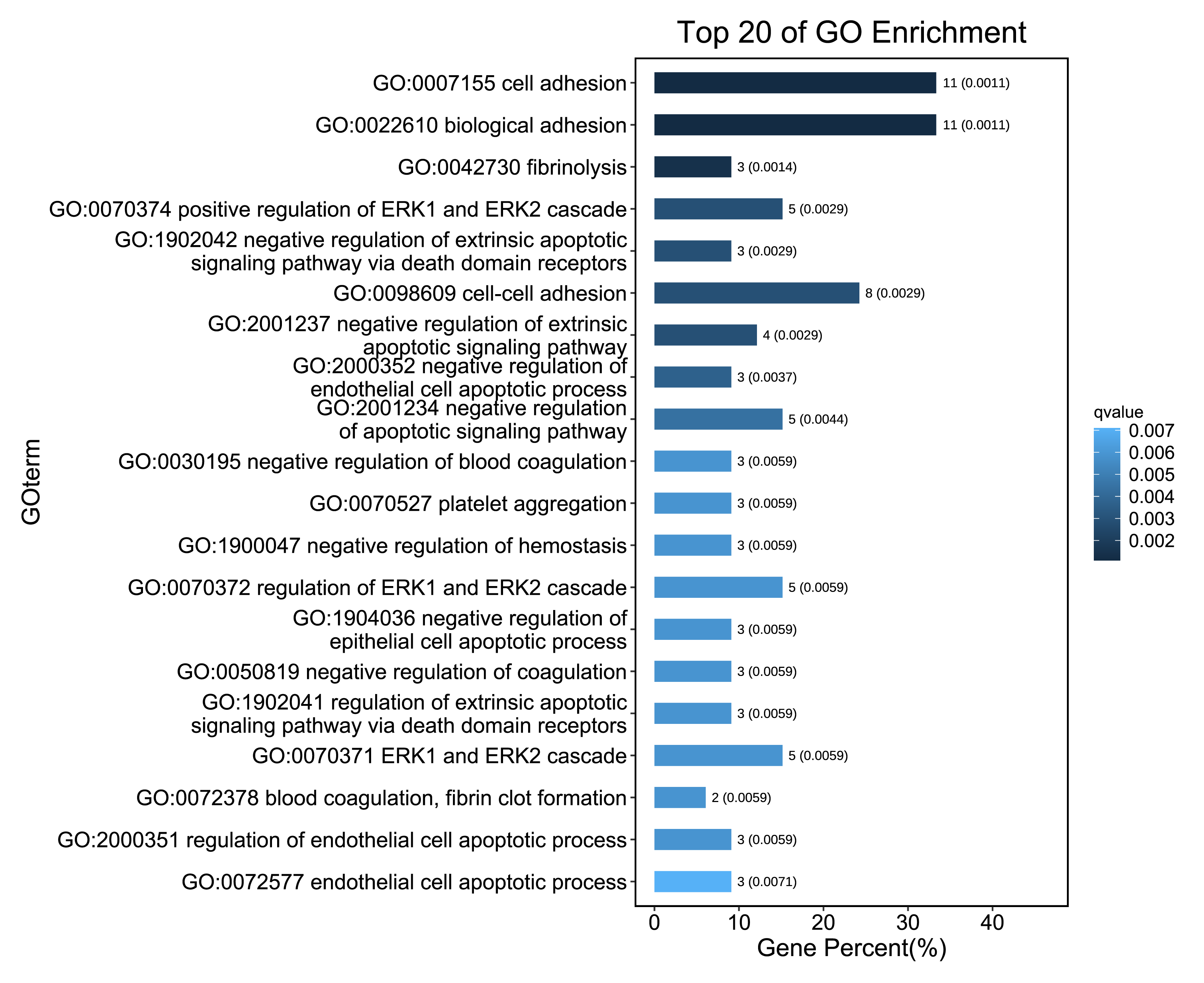

Supplement: Supplementary file 1 [file biology-11-01708-s001.zip › Supplementary Figure S7.png]
